# Peptides/receptors signaling during plant fertilization

**DOI:** 10.3389/fpls.2022.1090836

**Published:** 2022-12-15

**Authors:** Tian-Ying Yu, Chun-Xia Xu, Wen-Jia Li, Bo Wang

**Affiliations:** College of Life Sciences, Yantai University, Yantai, China

**Keywords:** double-fertilization, peptide, kinase, pollen tube guidance, polytubey block, cell-cell communication

## Abstract

Double fertilization is a unique and particularly complicated process for the generation alternation of angiosperms. Sperm cells of angiosperms lose the motility compared with that of gymnosperms. The sperm cells are passively carried and transported by the pollen tube for a long journey before targeting the ovule. Two sperm cells are released at the cleft between the egg and the central cell and fused with two female gametes to produce a zygote and endosperm, respectively, to accomplish the so-called double fertilization process. In this process, extensive communication and interaction occur between the male (pollen or pollen tube) and the female (ovule). It is suggested that small peptides and receptor kinases play critical roles in orchestrating this cell-cell communication. Here, we illuminate the understanding of phases in the process, such as pollen-stigma recognition, the hydration and germination of pollen grains, the growth, guidance, and rupture of tubes, the release of sperm cells, and the fusion of gametes, by reviewing increasing data recently. The roles of peptides and receptor kinases in signaling mechanisms underlying cell-cell communication were focused on, and directions of future studies were perspected in this review.

## Introduction

The life cycle alternates between the diploid sporophytic generation and the haploid gametophytic generation in flowering plants. Meiosis and double fertilization are indispensable and crucial programs for the alternation of generations (Hater et al., 2020; Hafidh and Honys, 2021). The mature gametophyte, the pollen grain, is a cell-in-cell structure, which means that two small sperm cells are embedded in the cytoplasm of a big vegetative cell. The polygonum-type embryo sac is found in the majority of flowering plants. It is a polarized structure with two synergids, one egg, one central cell that contains two nuclei, and three antipodals arranged from the micropyle to the chalazal end. In contrast to animal sperms, the immobile sperm cells of angiosperms are transmitted passively for long distances by pollen tubes that target the female gametes for double fertilization (Bleckmann et al., 2014). Germination and the subsequent elongation of the pollen tubes for transmission of sperm cells are initiated as the recognition of compatible grains and papillar cells. Pollen tubes penetrate the transmitting tract and are guided to the funiculus, then the micropyle of the ovule. The sperm cells will be released when the tube enters the embryo sac through the micropyle (Dresselhaus and Franklin-Tong, 2013). Complicated and extensive communication or interaction is involved during the process mentioned above. In the guiding signaling network, it is indicated that small peptide ligands and receptor kinases play essential roles (Chevalier et al., 2011; Kawashima and Berger, 2011; Dresselhaus et al., 2016; Zhou and Dresselhaus, 2019; Hafidh and Honys, 2021; Kim et al., 2021).

The plant receptor-like kinases (RLKs) are classified as the largest RLKs/Pelle family, with more than 610 and 1130 members in *Arabidopsis thaliana* and *Oryza sativa L.*, respectively (Shiu et al., 2004). RLKs are characterized into various subfamilies according to the different extracellular domains (ECDs), including Leucine-rich repeat RLKs, lysine motifs (LysM) RLKs (Franck et al., 2018a; Yu et al., 2021a), wall-associated kinases (WAKs), lectin RLKs, proline-rich receptor kinases (PERK), and others. There is also a class of co-receptors that are featured with basic structures similar to that of receptor kinases and facilitate the recognition of ligands (Kirkbride et al., 2005; Hohmann et al., 2017). Small peptides act as signals to bind to the ECDs of receptor kinases/co-receptors to induce multimerization and *trans*-phosphorylation, and further activate downstream signaling cascades. Currently, most of the small peptide signals identified in plant reproduction are small cysteine-rich peptides, defensin-like peptides, or rapid alkalinization factors (RALFs) (Okuda et al., 2009; Amien et al., 2010; Takeuchi and Higashiyama, 2012; Kanaoka, 2018; Somoza et al., 2021). Due to the remarkable diversity of peptide/receptor/co-receptor pairs, this communicating mechanism of peptide-receptor interaction is fundamental to many signaling pathways.

The communication of pollen-pistil during fertilization is involved in the five stages mentioned above. The signaling pathways orchestrated by peptide/receptor kinases in angiosperm fertilization are reviewed and discussed here. Peptides, receptors, and their functions are summarized in **Table 1**.

**Table 1:** peptides, receptors, functions during plant fertilization.

Process	Peptides	Peptides derived from	Receptors	Receptor localizationcell	Functions	Species	ref
Pollen-stigma recognition	SCR/SP11	anther tapetum cells	SRK MLKS THL1/2	papillae	Self-incompatible response	*Brassica*	Bower et al., 1996; Murase et al., 2004; Scandola and Samuel, 2019
	PCP-Bs	Pollen coat	FER ANJ HERK1	Papillae	pollen hydration and germination	*Arabidopsis*	Liu et al., 2021a; Wang et al., 2017; Galindo-Trigo et al., 2020
	RALF23 RALF33	papilla cells	FER ANJ LLG1	papillae	Maintenance of high levels of ROS in the stigma papillae	*Arabidopsis*	Liu et al., 2021a
	LAT52	anther	LePRK1/2	pollen	Inhibition of pollen germination before pollination	*Solanum Lycopersicum*	Tang et al., 2002; Johnson and Preuss, 2003
Pollen tubes growth in transmitting tract	LeSTIG1	stigma	LePRK1/2	pollen	Pollen tube growth	*Solanum Lycopersicum*	Tang et al., 2004; Zhang et al., 2008
	RALF4/19	Pollen tubes	BUPS1/2 ANX1/2 LLG2/3 MRI AUN1/2	Pollen tubes	Pollen tube growth and integrity	*Arabidopsis*	Boisson-Dernier et al., 2015; Ge et al., 2017; Ge et al., 2019; Franck, 2018b; Zhou et al., 2021
	RALF4	Pollen tubes	LRX8/9/10/11	Pollen tubes	Pollen tube growth and integrity	*Arabidopsis*	Mecchia et al., 2017; Fabrice et al., 2018
Pollen tubes guidance	TfLURE1/2	synergid cell	N.D.	N.D.	pollen tube guidance and attraction at the micropyle	*Torenia fournieri*	Okuda et al., 2009
	AtLURE1.1-1.5 AtLURE1.7 AtLURE1.8	synergid cell	PRK6 MDIS1/2 MIK1/2	Pollen tubes	Species-specific pollen tubes attraction and guidance in the septum and micropyle	*Arabidopsis*	Takeuchi and Higashiyama, 2012; Takeuchi and Higashiyama, 2016; Wang et al., 2016; Zhong et al., 2019; Liu et al., 2021b
	XIUQIU1/2 XIUQIU3/4	synergid cell	N.D.	N.D.	Species-indiscriminate pollen tubes attraction and guidance	*Arabidopsis*	Zhong et al., 2019; Liu et al., 2021b
	ZmEA1	egg apparatus	N.D.	N.D.	Pollen tubes attraction and guidance	*Zea Mays*	Marton et al., 2005; Marton et al., 2012
	RALF6/7/16/36/37	pollen tube	FER-ANJ-HERK1 LLG1	Transmission track and septum of pistil	Polyspermy block at the septum and the micropyle	*Arabidopsis*	Zhong et al., 2022
Pollen tube reception and rupture	RALF6/7/16/36/37	pollen tube	FER-ANJ-HERK1 LLG1 NORTIA	micropyle	Polyspermy block at the micropyle	*Arabidopsis*	Ju et al., 2021; Huck et al., 2003; Zhong et al., 2022
	RALF4/19	Pollen tubes	BUPS1/2 ANX1/2 LLG2/3	Pollen tubes	Pollen tube growth and integrity	*Arabidopsis*	Ge et al., 2017; Ge et al., 2019
	RALF34	inner integument	BUPS1/2 ANX1/2 LLG2/3	Pollen tubes	Pollen tubes rupture	*Arabidopsis*	Ge et al., 2017; Ge et al., 2019
	ZmES1-4	synergid cell	N.D.	N.D.	Pollen tube rupture and sperm release	*Zea Mays*	Cordts et al., 2001; Amien et al., 2010
Gamete fusion and double fertilization	EC1s	egg cell			Guidance of HAP2/GCS1 of sperm cells to the membrane and gametes fusion	*Arabidopsis*	Sprunck et al., 2012; Hamamura et al., 2012

### Recognition of pollen and stigma

**Figure 1:**
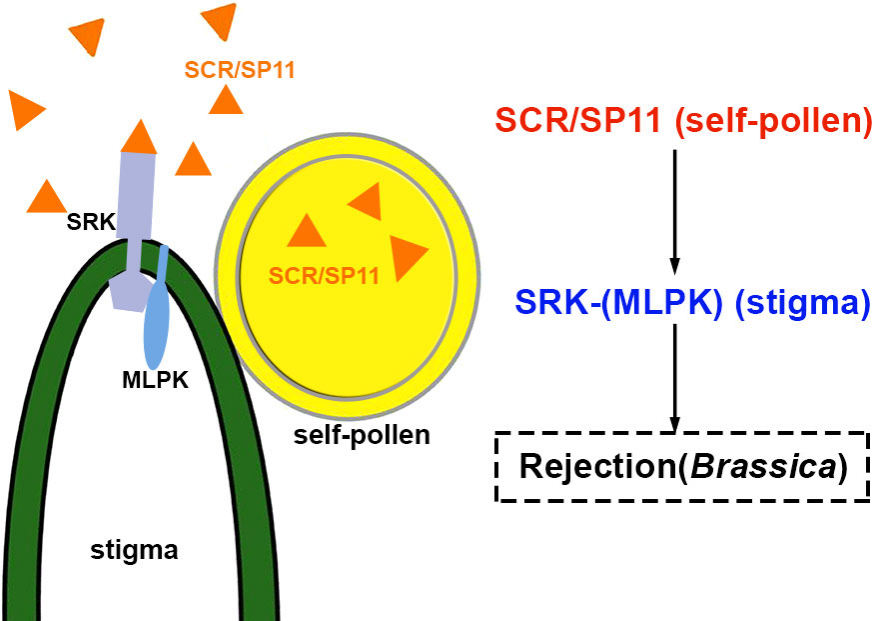
Schematic diagram of SCR/SP11 and SRK grouped for pollen-stigma interactions of *Brassica* self-incompatibility. The peptide triggering inhibition of signaling pathways is highlighted in red. The blue represents receptor complexes and the co-receptors are shown in parentheses. The source of peptides and receptors is shown in parentheses. Abbreviations for all small peptides and receptors are elucidated in the text.

The first checkpoint for plant fertilization is at the beginning of pollen-papilla interaction. The mature pollen grains (male gametophytes) land on the stigmatic papillae at the top of the pistil. The male and female are adhered to and recognized by each other. In self-incompatible plants, only the compatible grains can proceed with the subsequent hydration and germination (Rozier et al., 2020). The alien pollens from unrelated species or the self-incompatible (SI) ones are ineffective. In about half of the species in flowering plants, self-incompatibility and cross-pollination are applied to maintain genetic diversity within species populations (Murase et al., 2004; Zhang et al., 2021). Accumulating data indicate that this self/non-self-recognition in *Brassica* is triggered by *S*-haplotype-specific interaction between the male and female determinants. The *S*-haplotype consists of two or more closely linked polymorphic *S* loci. The pistil and pollen will show self-incompatibility given that the same *S* allele is present in the male and female. Rejection of pollen for the same individual *Brassica* plant is due to sporophytic self-incompatibility (Jany et al., 2019). The male determinant factor is a small peptide ligand, *S*-locus cysteine-rich protein/*S*-locus protein 11 (SCR/SP11), which is secreted from the anther tapetum cells, and diffused to the pollen wall and stored there. The female determinant factor is the *S*-locus receptor kinase (SRK) localized in the papillar cell membrane (Scandola and Samuel, 2019). The SI response is initiated by the haplotype-specific interaction between SCR/SP11 and SRK, the interaction through which the reciprocal communication between the pollen and papillar cells is regulated (Scandola and Samuel, 2019). SCR/SP11-SRK is involved in the self-incompatibility signal pathway in *Brassica*, as shown in **Figure 1**. In the stigma, SRK exists as a preassembled homodimer at the plasma membrane of papillae and remains inactive until pollination. SRK is activated by the *S*-haplotype-specific SCR/SP11 binding to its ECD and the autophosphorylation of its intracellular kinase. The intracellular serine/threonine M-LOCUS PROTEIN KINASEs (MLPKs) is anchored at the membrane of papillae, and acts as a downstream target for SRK (Murase et al., 2004). THL1 and THL2 are members of the thioredoxin-H family and function as negative effectors (Bower et al., 1996). The stigma-expressed E3 ubiquitin ligase ARMADILLO-REPEAT-CONTAINING1(ARC1) is a positive regulator in the pistil for the rejection of self-incompatible pollen in *Brassica napus*, *Arabidopsis lyrata*, and *Arabidopsis thaliana* (Stone et al., 2003; Goring et al., 2014). All these players mentioned above are involved in the self-incompatibility response.

In *Brassica*, in the absence of self-pollen, THL1/2 interacts with the intracellular domain of SRK to form a heterodimer that prevents SRK from homodimerization and activation (Bower et al., 1996; Mazzurco et al., 2001). In the presence of self-pollen, SCR/SP11 interacts with the ECD of SRK to promote SRK homodimerization and consequently results in the release of THL1 and THL2 from SRK. The active SRK recruits and phosphorylates MLPK (Murase et al., 2004; Liang and Zhou, 2018). The cytoplasmic kinase MLPK subsequently phosphorylates and activates ARC1 (Stone et al., 2003; Goring et al., 2014) to transduce the SI signaling. Phosphorylated ARC1 mediates ubiquitination and degradation of a series of downstream substrates involved in pollen hydration, exocytosis, and germination. For example, the factor for stigmatic compatibility: Exo70A1, is involved in pollen hydration (Kitashiba et al., 2011; Li et al., 2013; Zhang et al., 2016); the phospholipase D-1 protein (PLD1) plays role in germination (Scandola and Samuel, 2019). The glyoxalase 1 (GLO1) is required to detoxify plants’ methylglyoxal (MG) cytotoxicity. ARC1 was shown to ubiquitinate GLO1 effectively (Sankaranarayanan et al., 2015). In self-incompatible pollination, less GLO1 was found in stigma. It was suggested that the factors involved in compatible reactions were modified and disrupted by the enriched methylglyoxal. Eventually, the ‘self-pollen’ is rejected, and the papillae are degenerated (Sankaranarayanan et al., 2015).

In model plant *A. thaliana*, the acceptance of self-pollen by the stigma is attributed to the malfunction of self-incompatible genes *SCRs* and *SRKs* (Boggs et al., 2009). The *Brassica* pollen coat protein class B (PCP-B) are small cysteine-rich proteins (CRPs). Both PCP-Bs and SCRs are CRP proteins with eight conserved cysteines. However, it is shown that they belong to different small branches according to the phylogenetic analysis, and they are specialized for compatible pollination and self-incompatibility, respectively (Wang et al., 2017). In *A. thaliana*, defective pollen hydration and delay of tube growth are observed due to the disruption of genes for *PCP-Bs* (Wang et al., 2017). PCP-Bs serve as male factors for molecular dialogues of stigma—pollen to orchestrate pollen hydration, adhesion, and pollen tube growth (Wang et al., 2017). The reduction of reactive oxygen species (ROS) in papillar cells is closely correlated with the hydration and adhesion of pollen, especially at the adhesion sites (McInnis et al., 2006). When landed on the wild-type stigma, pollen from *pcp-bγ* and *pcp-bβ/γ* mutants showed markedly slow hydration because of severe suppression of the ROS reduction, in contrast to the response of wild-type pollen (Liu et al., 2021a). Wild-type stigmas treated with mature peptide PCP-Bγ exhibited a dose dependence on ROS reduction (Liu et al., 2021a). Fed with ROS inhibitor and ROS scavengers, the pistil with reduced ROS showed a significant acceleration of pollen hydration 10 minutes after pollination (Liu et al., 2021a).

The well-known receptor kinase FERONIA (FER) is expressed in various tissues and plays diverse essential roles (Escobar-Restrepo et al., 2007; Deslauriers and Larsen, 2010; Duan et al., 2010; Stegmann et al., 2017). In *Arabidopsis*, both receptor kinases ANJEA (ANJ) (Galindo-Trigo et al., 2020) and FER belong to *Catharanthus roseus* receptor-like kinase 1–like (CrRLK1L) family and are strongly expressed in stigma. The ANJ-FER receptor complexes act as essential female factors. They are involved in pollen—papilla communication by interacting with PCP-Bs for pollen hydration and germination (Liu et al., 2021a). When pollinated with wild-type pollen, rapid hydration phenotype was observed on the mutants pistil of *fer-4*, *anj-1*, and *fer-4 anj-1*, which was opposite to that of *pcp-bs* mutants. The phenotype was rescued in the complementation test (McInnis et al., 2006; Liu et al., 2021a).

The autocrine RALF23/33 peptides interact with the ECDs of FER and ANJ in stigma. Mutant *ralf33* showed a similar phenotype to *fer-4* and *anj-1* at reduced ROS accumulation in stigmatic papillae and accelerated pollen hydration (Liu et al., 2021a). LORELEI-like-GPI-anchored proteins (LLG)1, the co-receptor of FER-ANJ, is one of the members of the LORELEI (LRE) family (Feng et al., 2019). Loss of LLG1 function also resulted in reduced ROS accumulation in papillae. The guanine nucleotide exchange factors (GEFs) of Rho-like GTPases (RAC/ROP) and NADPH oxidases (RESPIRATORY BURST OXIDASE HOMOLOG D, RBOHD) act downstream of FER-ANJ-LLG1 to regulate ROS generation. Data from ROS-producing mutants suggested that autocrine RALF23/33 induces ROS generation through the ROP2-RBOHD pathway (Liu et al., 2021a).

**Figure 2:**
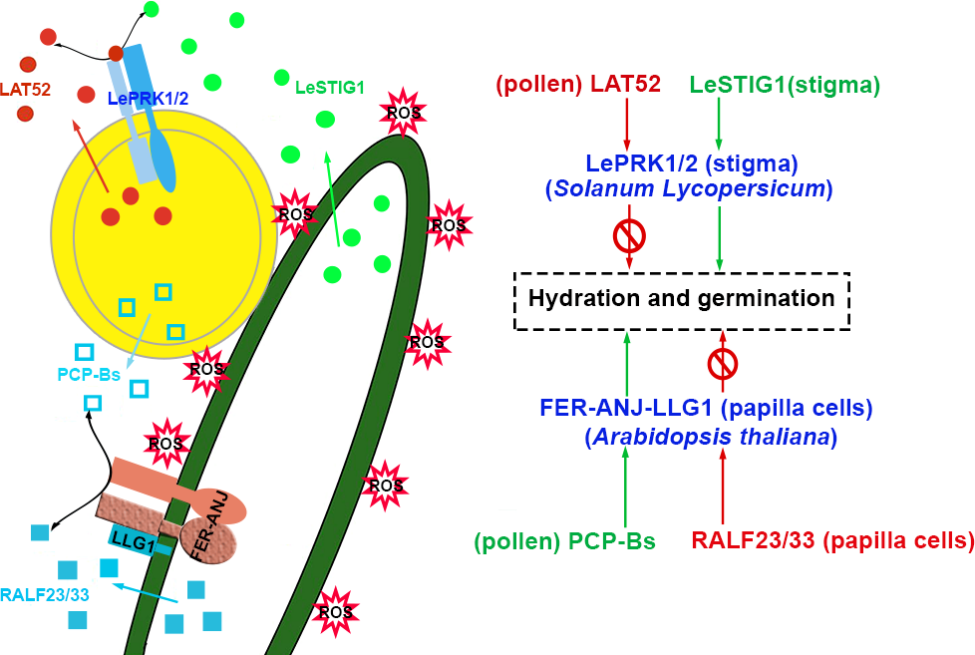
Pollen and stigma recognize each other to initiate the hydration and germination of pollen. In *Solanum Lycopersicum*, LAT52 interacts with LePRK1/2 to inhibit hydration and germination, and LeSTIG1 interacts with LePRK1/2 to facilitate hydration and germination. However, in *Arabidopsis*, pollen-specific PCP-Bs bind to FER/ANJ-LLG1 to promote hydration and germination, and stigmatic RALF23/33 interacts with FER/ANJ-LLG1 to maintain ROS accumulation and repress hydration of pollen. The peptides involved in the activation and inhibition of signaling pathways are highlighted in green and red, respectively. The receptor complexes are marked in blue and the co-receptors are shown in parentheses. The source of peptides and receptors is shown in parentheses. Abbreviations for all small peptides and receptors are elucidated in the text.

The dissociation constants (*K_d_*) of PCP-Bγ and RALF33 binding FER ectodomain (FERecd) were 0.34 μM and 0.1604 μM, respectively (Liu et al., 2021a). It means that RALF33 has a greater affinity for FERecd than PCP-Bγ does. However, the inhibition constant (*K_i_*) that PCP-Bγ competed to bind FERecd in the mixture of RALF33-FERecd was 2.5099 μM (Liu et al., 2021a). It seems that the results are somewhat contradictory. Without compatible pollen, RALF23 and RALF33 are secreted from stigmatic papillary cells and facilitate ANJ-FER and its co-receptor LLG1 to form polymer complexes that stimulate ROP2 to trigger RBOHD activity, which leads to maintaining the high level of ROS in papillae before pollination. (Liu et al., 2021a; Kou et al., 2022). It was proposed that after the landing of compatible pollen grains on the stigma, the pollen coat protein PCP-Bs competed with and replaced RALF23/33 to interact with the ANJ-FER receptor complexes in papilla cells. Consequently, the RALF23/33-initiated ROS signaling pathway was shut down. The ROS decreased rapidly in papillae to promote the hydration and germination of pollen grains. The maintenance of ROS content in papillar cells might be regulated through distinct signaling pathways stimulated by autocrine and paracrine peptides. The model is shown in **Figure 2**. These speculations need to be supported and verified by further experimental data.

Coincidentally, ligands exchange also occurs in the process of pollen growth and germination in tomato. The signaling pathway is triggered by receptor kinases at the pollen surface but not from female tissue. In *Solanum Lycopersicum*, cysteine-rich late-anther tomato 52 (LAT52), the male player, interacts with pollen-specific receptor kinase 2 (LePRK2) and prevents pollen from germination before pollination (Tang et al., 2002; Johnson and Preuss, 2003). After germination, another cysteine-rich peptide, stigma-specific protein 1 (LeSTIG1), is secreted and matured to a 7-kD peptide as the female player. LeSTIG1 accumulates on the pollen tube surface and binds the LePRK1/2 receptor complexes to promote pollen tube growth (Tang et al., 2004; Zhang et al., 2008; Huang et al., 2014). The ligand of the LePRK1/2 complexes, LAT52, is substituted by LeSTIG1. The signals could be switched from repression to activation of germination and tube growth, as shown in **Figure 2** (Tang et al., 2004). LeSTIG1-LePRK2 orchestrates ROS levels through phosphatidylinositol 3-phosphate PI(3)P signaling to promote pollen tube growth (Huang et al., 2014). The Kinase partner protein (KPP) of *S. lycopersicum* is a Rop-GEF that interacts with the cytoplasmic kinase domains of LePRK1 and LePRK2 through its C-terminus. It recruits Actin-Related Protein 2/3 (ARP2/3, actin nucleators) complexes to the membrane of the pollen tube tip and coordinates actin and cytoskeleton to enhance tube growth (Huang et al., 2014; Liu et al., 2020). It may also be in an essential manner through which tomato LeSTIG1 regulates the growth of pollen tubes in the transmitting tract. It is required to investigate whether LeSTIG1 is strongly expressed and localized in the stylar transmitting tract.

### Pollen tubes grow in the transmitting tract

In *Rosaceae*’s Gametophytic Self-Incompatibility (GSI) pollination, the pollen germinates and grows on the stigma. The growth of pollen tube arrests at one-third distance through the stylar transmitting tract (Franklin-Tong and Franklin, 2003). The GSI of *Rosaceae* is jointly regulated by the products of *S* locus, *S*-RNase of pistil determinants and *S*-haplotype specific F-box proteins/*S* locus F-box brothers (SFB/SFBB) of pollen determinant (Sassa, 2016; Sun et al., 2018; Matsumoto and Tao, 2019). In the transmitting tract, pistil *S*-RNases penetrate the pollen tube to interact with the male determinants. Pollen SFB/SFBB recognizes each a subset of non-self *S*-RNases, ubiquitinates them, and mediates their degradation by 26S proteasome (Entani et al., 2014). Self-*S*-RNase disrupted tip-localized ROS and decreased the Ca^2+^ current, inducing nuclear DNA degradation and pollen tube disintegration (Wang and Kao, 2012; Del Duca et al., 2019).

The compatible pollen germinates, penetrates the papillae (Iwano et al., 2014), and continues to grow through the transmitting tract. The pollen tubes elongate considerably in style, and their integrity is maintained to prevent premature rupture. There are at least two signaling networks for the integrity of the pollen tube in the transmitting tract and the burst upon arrival at its destination.

RALF is a class of small peptide ligands widely known in various signaling pathways. The pollen tube-specific tomato (*S. lycopersicum*) RALF (SlPRALF) small peptides are responsible for pollen tube growth in different developmental windows (Covey et al., 2010). Eight pollen specifically expressed RALFs are clustered into three phylogenetic clades among the 37 RALFs in *A. thaliana*. RALF4 and RALF19 are grouped into one clade. The pollen tube of the homozygous and pollen-specific-promoter-*amiRRALF4/19* transgenic plants fails to grow in the transmitting tract and cannot reach the ovule *in vivo* (Mecchia et al., 2017). Nearly 70% of pollen tubes from the *amiRRALF4/19* plants burst *in vitro* germination of pollen tube assay (Mecchia et al., 2017). In the *ralf4-1*mutant, fertilization could occur, but 47% of pollen tubes burst *in vitro* germination (Mecchia et al., 2017). It is suggested that RALF4/19 plays a crucial and redundant role for pollen tube integrity and growth. Disruption of ANXUR1(ANX) and ANXUR2, the closest members to FERONIA in the CrRLK1L subfamily, phenocopies *amiRRALF4/19* lines in pollen tube dehiscence and elongation repression (Boisson-Dernier et al., 2009; Miyazaki et al., 2009). The rupture of tubes in *amiRRALF4/19* cannot be rescued with overexpression of *ANX1/2* (Mecchia et al., 2017). It is indicated that ANX1/2 is the downstream receptor of RALFs, which might coordinate other components to regulate tube integrity.

Meanwhile, Mecchia et al., 2017 found that premature tube rupture resulted from the repression of pollen-specific ANX1 and ANX2 *in vitro* germination assay. A similar phenotype was also observed when *BUDDHA’S PAPER SEAL (BUPS) 1/2* was knocked out (Ge et al., 2017). The T-DNA or knock-down mutant of *bups1* showed apical rupture after exiting the style and cessation of growth at the apex of the transmission tract (Ge et al., 2017). It is demonstrated that the mutants failed to maintain the integrity of the pollen tube. BUPS1 is involved in the perception and response to mechanical stress when tubes pass through style. It is suggested that BUPS1 activates ROP1 GTPase signals directly to promote exocytosis that accelerates BUPS1-dependent secretion of RALF4 (Ge et al., 2019; Kou et al., 2022). RALF4, acting as a cognate ligand for BUPS1, activates BUPS1 to amplify signaling and strengthen the rigidification of the cell wall (Zhou et al., 2021). It is proposed that these four receptor kinases are involved in maintaining pollen tube integrity in *Arabidopsis*.

The pollen-tube-expressed glycosylphosphatidylinositol-anchored proteins (GPI-APs), LORELEI-like-GPI-anchored protein 2/3 (LLG2/3), are engaged in maintaining tube integrity. Early rupture of pollen tubes and severe fertility defects result from *LLG2/3* disruption (Ge et al., 2019). LLG2/3 interacts with the extracellular domains of BUPS1/2 and ANX1/2 *in vitro* pull-down assay, and the interaction is enhanced by RALF4/19 (Ge et al., 2019). It is suggested that LLG2 and LLG3 act as co-receptors of the BUPSs/ANXs to regulate pollen tube integrity. RALF4 interacts with LLG3 and BUPSs/ANXs *via* different N-terminal domains. The N-terminal YISY motif of RALF4 is independent of the interaction but responsible for pollen tube integrity (Ge et al., 2019). It means that other proteins or receptors may interact with the N-terminal YISY domain of RALF4, which plays an essential role in pollen tube integrity in *Arabidopsis*.

LRX (LEUCINE-RICH REPEAT EXTENSIN) is the partner of RALFs in tomato. The pollen-specific LRX8/9/10/11 regulates the integrity of pollen tubes in *Arabidopsis* (Mecchia et al., 2017; Fabrice et al., 2018). LRXs play critical roles in maintaining cell wall rigidity during pollen tube growth and play essential roles in physical links between the intra- and extracellular components. The *lrx* multiple mutants showed premature rupture of pollen tubes and sterility to different extents (Mecchia et al., 2017). This phenotype was similar to that of mutants with the disruption of *BUPSs* and *ANXs*, and could not respond to the RALF4-triggered pollen tube growth. LRXs are linked through disulfide bonds to form dimers, and their N-terminal region physically interacts with RALF4 (Mecchia et al., 2017; Fabrice et al., 2018). RALF4/19 directly interacted with ANX1/2-BUPS1/2 to orchestrate the growth and integrity of pollen tubes (Ge et al., 2017). In addition, the N-terminal domains of LRX1/2/4/5 were discovered to interact with FER in vegetative tissues physically (Herger et al., 2020). It is very significant to explore whether LRX8/9/10/11 interacts with the ANX1/2-BUPS1/2 complexes in maintaining the integrity of pollen tubes. It is unknown whether LRXs interact with RALF4 through the N-terminal YISY motif of RALF4.

The coordination between RALF4-LRX8 and RALF4/19-ANX1/2-BUPS1/2 was identified *via* the downstream factors of the signaling cascade. The *mri-3D* was obtained in the *anx1anx2* mutagenesis screening for repression of pollen tube rupture (Boisson-Dernier et al., 2015). Due to the R240C nonsynonymous replacement in the kinase activation loop of MARIS (MRI) resulting in excessive kinase activity, *mri-3D* showed the opposite phenotype. MRI is a member of the receptor-like cytoplasmic kinases (RLCKs) localized at the plasma membrane. MRI functions as the downstream player of ANX1/ANX2 to maintain the integrity of pollen tubes (Boisson-Dernier et al., 2015). ANX1/2 acts upstream of NADPH oxidase (RESPIRATORY BURST OXIDASE HOMOLOG H/J, RBOHH/RBOHJ) of the pollen tube membrane and affects the matrix exocytosis of cell wall for the tube integrity. NADPH oxidase generates tip-accumulated ROS to maintain the calcium gradient at the apex, ultimately regulating the growth and integrity of pollen tubes (Boisson-Dernier et al., 2013). MRI^R240C^ displayed hyperactive and could partially rescue the phenotype of pollen tubes burst of *anx1anx2* (Boisson-Dernier et al., 2015). It is proposed that MRI might function downstream of RBOHH/J. It is shown that both ANX1 and MRI cannot rescue the rupture of pollen tubes of *amiRRALF4/19* when ANX1-YFP and MRI-YFP are exclusively expressed in pollen tubes (Mecchia et al., 2017). Therefore, it is proved that two parallel signaling pathways are activated by the cysteine-rich peptides RALF4/19 that are secreted by pollen tubes.

The protein phosphatase ATUNIS1/2 (AUN1/2) is discovered to act downstream of ANX1/2 *via* screening for suppression to untimely rupture of the *anx1anx2* pollen tube (Franck et al., 2018b). However, it is the activity of AUN1^D94N^, but not the expression of MRI^R240C^, sufficient to attenuate male sterility and pollen burst phenotype in *lrx8-11* quadruple mutants (Franck et al., 2018b). It is suggested that AUN1/2 phosphatases may equilibrate MRI kinase activity and regulate the phosphorylation status of target substrates for the growth and integrity of the tubes (Vogler et al., 2019).

**Figure 3:**
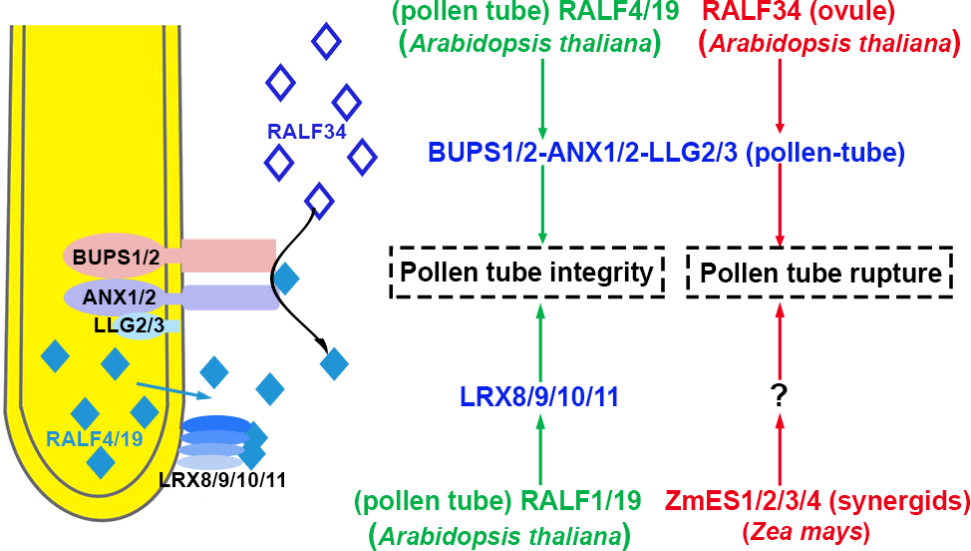
The integrity of pollen tube growth is maintained, and pollen tubes rupture In *Arabidopsis thaliana*, pollen-specific RALF4/19 orchestrates pollen tube receptor complexes BUPS1/2-ANX1/2-LLG2/3 and LRX8/9/10/11 to maintain pollen tube growth and integrity in the transmitting tract. After the pollen tube enters the ovule, the ovule-specific RALF34 interacts with BUPS1/2-ANX1/2-LLG2/3, resulting in pollen tube rupture. In *Zea Mays*, ZmES1/2/3/4 secreted from synergid cells causes pollen tube rupture. The peptides involved in the activation and inhibition of signaling pathways are highlighted in green and red, respectively. The blue represents receptor complexes and the co-receptors are shown in parentheses. Question marks indicate unidentified signal components. The source of peptides and receptors is shown in parentheses. Abbreviations for all small peptides and receptors are elucidated in the text.

Therefore, the integrity of pollen tubes is regulated jointly by parallel pathways, RALF4/19-LRX8/9/10/11-AUN1/2 and RALF4/19-ANXs/BUPSs-MRI signaling pathway, as shown in **Figure 3**. The phosphorylation status of downstream substrates is equilibrated and maintained by negative regulator AUN1/2 *via* the RALF4/19-LRX8/9/10/11-AUN1/2 signaling pathway. The positive effector MRI facilitates the phosphorylation of downstream targets by RALF4/19-ANXs/BUPSs-MRI pathway (Ge et al., 2017; Mecchia et al., 2017). However, RALF4/19 is allocated by an unknown mechanism. As mentioned previously, BUPS1-activated ROP1 promotes BUPS1-dependent secretion of RALF4. It was suggested that RALF4/19 triggered ANX1/2-BUPS1/2-MRI, and activated the kinase activity of MRI. Simultaneously, RALF4/19 activated LRX8/9/10/11-AUX1/2 signaling pathway, exerting the phosphatase activity of AUX1/2 to balance MRI kinase activity. These two pathways jointly coordinated and regulated tube growth and integrity.

It is suggested that the abundant extracellular matrix protein, arabinogalactan protein AGP is involved in tube elongation in the transmitting tract (Leszczuk et al., 2019). AGP is a hydroxyproline-rich glycoprotein that can adhere to the surface of the pollen tube. AGP can be integrated into the cell wall matrix of the pollen tubes and decrease after hydrolyzation by deglycosylase (Coimbra et al., 2008; Jia et al., 2015). Continuous adhesion and integration of AGP are necessary to promote tube elongation. The slow growth of pollen tubes resulted from decreased AGPs in the surface of pollen tubes from *Arabidopsis agp6 agp11* mutants (Levitin et al., 2008; Costa et al., 2013). In addition, the growth and elongation of pollen tubes are also associated with the glutamate-derived signaling molecule GABA (Ramesh et al., 2015). Low-concentration GABA stimulates pollen tube growth *in vitro*; however, gradually increasing GABA is required for tube guidance in the transmission tract (Ma, 2003; Palanivelu et al., 2003). The role of AGP and GABA, especially AGP, as physiological signals still needs to be determined.

### Pollen tube guidance and polytubey block

Chemoattractants emitted by the ovule (especially synergids) direct the growth of the pollen tube along the funiculus towards the micropyle. In *Torenia fournieri* (*Linden. ex Fourn*), four cells in a mature embryo sac, including a central cell, an egg cell, and two synergid cells, were ablated individually or in groups. The capability of pollen tube attraction is lost in the ovule with two ablated synergids (Higashiyama et al., 2001). It indicates that the synergid cell produces signals to attract the pollen tube growth toward the ovule. The synergid cells from *T. fournieri* were isolated and studied to identify the chemoattractant. A class of small cysteine-rich defensin-like peptides, abundantly and predominantly expressed in synergid cells, were determined. Mature peptides of LUREs (LURE1 and LURE2) produced in *E. coli* showed guiding activity for pollen tube targeting *in vitro* assay for pollen tube attraction (Okuda et al., 2009). AtLURE1 peptides were secreted merely from synergid cells and diffused along the funicular surface, guiding the growth of pollen tubes to micropyle (Takeuchi and Higashiyama, 2012). The AtLURE1s*’* variants were inefficient in reorienting the pollen tube under the semi-*in vivo* pollen tube attraction assay (Zhong et al., 2019). Mutants of 23 pollen-specific receptor kinases were used to study the attraction capability of the AtLURE1s peptide. Pollen tubes from *prk6* ignored attractants and could not be redirected toward AtLRUE1.2 (Takeuchi and Higashiyama, 2016). PRK6 served as a vital receptor for AtLURE1 signals *in vitro* attraction assay. PRK6-mRuby2 was asymmetrically localized at the surface of pollen tubes, with the attraction of AtLURE1.2. It was suggested that PRK6 acted as the male player to sense AtLURE1s signaling from synergid cells to guide pollen tube growth toward the micropyle (Takeuchi and Higashiyama, 2016).

Lost in Pollen tube guidance 1 and 2 (LIP1 and LIP2) are cytoplasmic receptor kinases localized at the membrane of tube tips through palmitoylation at the N-terminal cysteine site. *In vivo*, 46% of *lip1 lip2* pollen tubes showed guidance defects to the micropyle. The transmitting effciency of male gametes was 43% in *lip1 lip2*. Thirty percent of *lip1 lip2* pollen tubes were insensitive to the female attractant AtLURE1.2 *in vitro* attraction assay, although 95% of the wild-type pollen tubes were attracted (Liu et al., 2013). LIP1/2 interacted with tip-localized PRK6 and was involved in response to the AtLURE1s (Liu et al., 2013). It was demonstrated that LIP1 and LIP2 were not necessary components of the PRK6 receptor complexes in micropylar guidance signaling pathways (Liu et al., 2013; Takeuchi and Higashiyama, 2016).

Wang et al. reported that LUREs specifically interacted with the ECDs of three receptor kinases, MALE DISCOVERER1(MDIS1), MDIS1-INTERACTING RECEPTORS LIKE KINASE1 (MIK1), and MIK2, to attract pollen tubes in a self-favorite way (Wang et al., 2016). PRK6 contains six LRRs in its ectodomain to form a solenoid structure. AtLURE1s, which act as female participants of pollen tube guidance, bind directly to the juxta-membrane region of PRK6, despite the extracellular LRR domains that interact specifically with ligands (Takeuchi and Higashiyama, 2016). It was proposed that other ligands might interact with the LRR domain of PRK6 to initiate pollen tube guidance. In addition to PRK6, MDIS1/MIK1/MIK2 acted as other receptors for AtLURE1s.

LUREs’ attraction to pollen tubes was based on conspecific preferential principles so that the interspecific genetic isolation and maintaining of species specificity are promoted. Recombinant AtLURE1.3 peptides preferentially attracted *A. thaliana* but not *A. lyrata* pollen tubes *in vitro* (Takeuchi and Higashiyama, 2012; Zhong et al., 2019). Consistently, AtLURE1 expressed in *T. fournieri* synergids fascinated *A.thaliana* pollen tubes to *T. fournieri* ovule (Takeuchi and Higashiyama, 2012). It is suggested that LURE1s are species-specific pollen attractants for interspecies specificity and reproductive isolation. It implies that additional and common pollen-tube attractants might be released from ovules.

**Figure 4:**
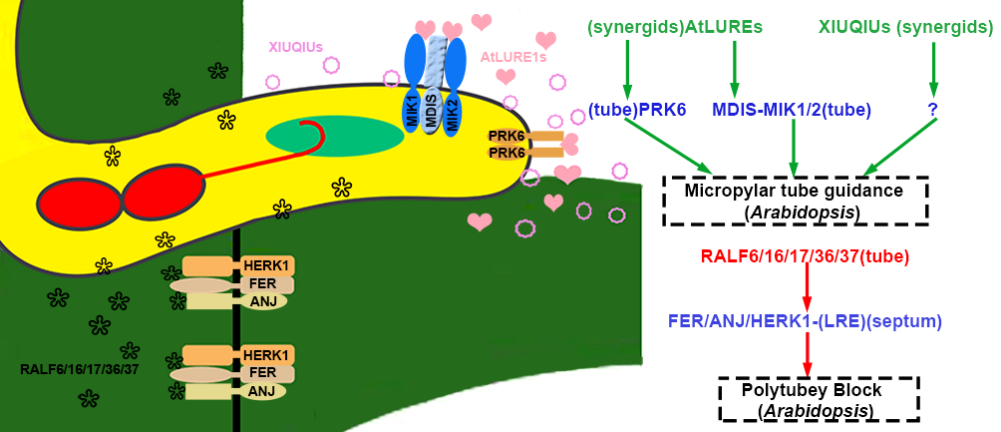
The micropyle guidance and polytubey block of pollen tubes. In *Arabidopsis thaliana*, pollen tubes penetrate the septum, and the RALF6/16/17/36/37 secreted by the pollen tube interacts with the septum-specific FER/ANJ/HERK1 to initiate the polytubey block. XIUQIUs and AtLURE1s secreted by synergid cells diffuse along the funiculus. AtLURE1s interact with PRK6 and/or MIK1/2-MDIS of the pollen tube to guide the pollen tube to grow towards the micropyle. The peptides are highlighted in green and red to represent activation or suppression, respectively. The blue represents receptor complexes and the co-receptors are shown in parentheses. Question marks indicate unidentified signal components. The source of peptides and receptors is shown in parentheses. Abbreviations for all small peptides and receptors are elucidated in the text.

In addition, loss-of-function *atlure1* septuple mutant and null *prk6* showed natural fertility (Zhong et al., 2019). The *A. lyrata* pollen tubes were attracted to pass through the septum to the micropyles of ovules in null *atlure1* pistil. It is proposed that general genus attractants are present in *atlure1* ovules. The XIUQIUs, a close relative of AtLURE1 in *Brassicaceae*, are small cysteine-rich peptides secreted from synergid cells to accumulate near the filiform apparatus. The recombinant XIUQIUs indiscriminately attract the pollen tubes of *A.thaliana* and *A. lyrata* under semi-*in vivo* assay for pollen tube attraction (Zhong et al., 2019). XIUQIUs take roles as female players, and their pollen tube-localized receptors still need to be identified. Compared with non-species-specific XIUQIUs, AtLURE1s-PRK6 and AtLURE1s-MDIS1/MIK1/MIK2 control conspecific pollen tube-preferred signaling pathways at septum and micropyle, as shown in **Figure 4** (Liu et al., 2021b).

Besides XIUQIUs and LUREs, there are 73 other CRP proteins expressed exclusively in synergid cells of *Arabidopsis*, and the roles of these CRPs are still unknown (Takeuchi and Higashiyama, 2012). The mutants showed an abortion rate of 20% in the seed sets after the knockout of all clade genes (CRP810), including XIUQIUs and LUREs (Liu et al., 2021b). It is suggested that attraction for the oriented growth of pollen tubes is lost incompletely. It indicates that there must be additional attractants of pollen tubes in synergids or other female gametophytic cells (Hater et al., 2020).

For instance, the capability of pollen tubes for chemotropic response to LURE is induced and enhanced by methyl-glucuronosyl arabinogalactan (AMOR) in *T. fournieri* ovules (Mizukami et al., 2016). As tube guiding attractants in maize, ZmEA1 is exclusively expressed in the egg apparatus, subsequently diffused toward the filiform apparatus, and finally localized at the nucellar cell wall below the micropylar end (Marton et al., 2005; Marton et al., 2012). The pollen tube-specific cognate receptor for the ZmEA1 has yet to be discovered. Coincidentally, the glycoproteins secreted into the obturator pore of the apple embryo sac were reported to direct the pollen tube toward the ovule (*Malus × domestica*) (Losada and Herrero, 2017).

**Figure 5:**
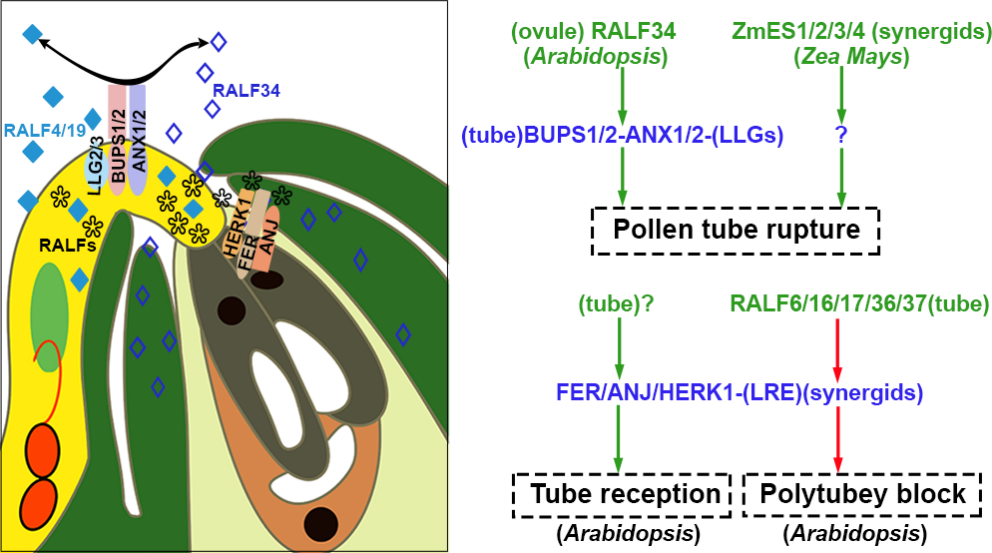
Pollen tube is received, polytubey block, and rupture. At the micropyle, FER/ANJ/HERK1-LLG1 derived from synergid cells is involved in the recognition and reception of pollen tube; they also interact with tube-specific RALF6/16/17/36/37 to cause polytubey block. After the pollen tube enters the ovule, the interaction between ovule-specific RALF34 and BUPS1/2-ANX1/2-LLGs of the pollen tube results in the rupture of the tube in *Arabidopsis*. ZmES1/2/3/4 secreted by synergids is related to the rupture of the pollen tube in *Zea Mays*. The peptides are highlighted in green and red to represent activation or suppression, respectively. The receptor complexes are marked in blue and the co-receptors are shown in parentheses. Question marks indicate unidentified signal components. The source of peptides and receptors is shown in parentheses. Abbreviations for all small peptides and receptors are elucidated in the text.

Polytubey block occurs at the micropyle and the pistil’s septum. It is indispensable to ensure that only one conspecific pollen tube passes through the septum and grows along the funiculus to target the micropyle. GENERATIVE CELL SPECIFIC 1 (GCS1)/HAPLESS 2 (HAP2) is specifically expressed in sperm cells and plays a vital role in membrane fusion during double fertilization (Mori et al., 2006; von Besser et al., 2006). In null *hap2*, the double fertilization failure resulted from impaired membrane fusion. However, fertilization recovery is successful when the second pollen tube comes 7 hours after pollination. However, multiple pollen tubes crossed out of the septum for fertilization recovery since the polytubey block was disrupted in mutants. RNA-seq data from the transmission tract and septum of the pistil indicated that FER, ANJEA (ANJ), and HERCULES RECEPTOR KINASE 1 (HERK1), malectin-like domain-containing receptor-like kinase (MLD-RLK) (also known as *C. roseus* RLK1-LIKE or CrRLK1L), were highly expressed. Multiple pollen tubes grew along the funiculus 5 hrs after pollination in the pistils of *fer-4*, *anj herk1*, or *fer anj herk1* (Zhong et al., 2022). These three CrRLK1L receptor kinases functioned as female players to communicate with pollen tube signals. In pollen-specific MYB mutants, *myb97myb101myb120*, the polytubey phenotype similar to the three null receptors was identified. The expression profiling data demonstrated that five *RALFs* (*RALF6*, *RALF7*, *RALF16*, *RALF36*, and *RALF 37*) were downregulated. Mutants of multiple combinations of *RALFs* knockout exhibited polytubey when pollinated to wild-type pistil. It is indicated that five RALFs (pollen tube-specific RALF6/7/16/36/37 peptides) function as ligands to be perceived and interact with the FER-ANJ-HERK1 receptor complexes (Zhong et al., 2022). It was speculated that a polyspermy block might be established through RALFs-FER/ANJ/HERK1 at the septum until tubes burst in the ovule after the removal of RALFs signaling as shown in **Figures 4**, **5**. Polyspermy block initiated upon the exit of pollen tubes from the septum and ended up at the vanishing of RALFs signaling after tube discharge. The mechanisms underlying polytubey block might play roles in the recognition between pollen tubes and synergids. The particular block should be precisely restricted in an appropriate spatial and temporal window. If fertilization fails, the persisting synergid cell would be responsible for secreting signals to attract additional pollen tubes for fertilization recovery.

### Discharge of sperm cells from pollen tubes

After arrival at the micropyle, the pollen tube is recognized by one of the two synergid cells, and the following communication occurs. Subsequently, the synergid cell undergoes programmed cell death, and the tube bursts to release two sperm cells. Two sperms fuse with two female gametes to form a zygote and primary endosperm, respectively. The persistent synergid cell undergoes degeneration and nourishes primary endosperm, preventing the attraction of excessive pollen tubes. Pollen tube reception and growth termination are tightly controlled by cell-cell communication between pollen tubes and synergid cells. The rupture of pollen tubes is also elaborately regulated through molecular communication of signals from the inner integuments and pollen tubes.

In *fer*, two or more pollen tubes enter a single ovule. It is demonstrated that the *fer* female gametophyte keeps attracting more tubes to enter the same ovule after the reception of the first tube. The polyspermy block mechanism is ineffective in *fer* (Pereira et al., 2016b). FER is localized at the membrane of the filiform apparatus and functions as receptor kinase to sense peptide signals in cell-cell communication. FER participates in receiving the first pollen tube and hinders extra tubes from polytubey and polyspermy (Huck et al., 2003; Escobar-Restrepo et al., 2007). The overgrowth of pollen tubes occurs when the A.thaliana fer is pollinated with pollen from A. lyrata or C. flexuosa (Escobar-Restrepo et al., 2007). It is suggested that FER plays an essential role in receiving signals from conspecific pollen tubes to maintain reproductive barriers.

HERK1 and ANJ are homologs of FER and localized in the filiform apparatus of the synergids. In *anj herk1*, a similar phenotype of tube overgrowth and failure of fertilization was identified, indicating that HERK1 and ANJ were functionally redundant regulators for pollen tube reception. (Galindo-Trigo et al., 2020). In *fer anj herk1*, pollen tubes were not able to be recognized and overgrown at the surface of the ovule, resulting in various rates of seed set abortion (Zhong et al., 2022). It was concluded that HERK1 and ANJ, as well as FER, served as the female participants for pollen tube reception and took roles in polytubey block at the micropyle. As pollen from different *ralfs* (*ralf36 ralf37, and ralf6 ralf7 ralf16 ralf36 ralf37*) were pollinated to the stigma of wild type, the phenotype of pollen tube overgrowth and seed set abortion were more severe as the increase of the disrupted RALFs. The phenotype was similar to that of *fer anj herk1* (Zhong et al., 2022). The data were consistent with the physical interaction of RALFs with FER-ANJ-HERK1 in an *in vitro* pull-down assay.

The co-receptor LORELEI (LRE) is involved in the interaction between RALF6/7/16/36/37 and FER-ANJ-HERK1. FER-ANJ-HERK1 received pollen tube signals at the micropyle, leading to polar accumulation of the downstream NORTIA (MLO protein) at filiform apparatus. Conversely, NORTIA promotes the perception of pollen tubes to ensure the polytubey block for double fertilization (Ju et al., 2021).

Now it is known that mechanisms underlying the recognition of pollen tubes at the septum and filiform apparatus are triggered by the above players coordinately. RALFs and FER-ANJ-HERK1 take critical roles in the reception of pollen tubes at the filiform apparatus of synergids and act as the second tier of the polytubey barrier as shown in **Figures 4**, **5** (Zhong et al., 2022). Once the pollen tube rupture in the ovule, the RALFs derived from tubes are diluted and diffuse into embryo sac. The interaction between RALFs and FER-ANJ-HERK1 receptor complexes is disrupted, so the polytubey barrier controlled by RALFs-FER/ANJ/HERK1 is removed at the septum and micropyle.

RNAi knock-down of *ZmES4* or *in vitro* treatment with ZmES4 (Zea mays Embryo Sac), the polymorphic defensin-like cysteine-rich protein from maize, induced tube rupture to release sperm cells in a species-preferred manner, as presented in **Figure 5** (Amien et al., 2010). ZmES4 is accumulated in vesicles of mature synergid cells and released upon the arrival of pollen tubes. The subsequent interaction between ZmES4 and KZM1(K^+^ channel Zea mays 1) leads to channel opening, K^+^ influxion, plasma membrane depolarization, water uptake, and eventual osmotic pollen tube burst (Cordts et al., 2001; Philippar et al., 2003; Amien et al., 2010).

It was speculated that the pollen tube entered the degenerated synergid cell and continued to grow and release sperm cells at the cleft between the egg and central cell. Another class of ligands should shut down the signaling pathways mentioned above for pollen tube integrity and growth. Pollen-tube localized receptors ANX1/2-BUPS1/2 would perceive RALF4/19 peptides secreted from pollen to regulate the tube growth integrity (Ge et al., 2017; Mecchia et al., 2017). However, an antagonistic signal would be required to disengage signaling pathways triggered by RALF4/19-ANX1/2-BUPS1/2 in ovules. This signal for the rupture of pollen tubes might be derived from the female tissue and received by receptors on the pollen tubes.

In contrast to RALF4, required for maintaining pollen tube integrity, a low concentration (2nM) of RALF34 caused the rupture of 23% of pollen tubes, and almost 70% of tubes are ruptured when treated with 20 mM RALF34 (Ge et al., 2017). Before fertilization, RALF34 accumulates in the ovule, especially in the inner integument. After the pollen tube’s arrival, it diffuses toward the micropyle/synergid cell region. The *ralf34* mutant did not display fertility defects, suggesting that other RALFs in female reproductive tissue are involved in the burst of pollen tubes and release of sperm cells (Ge et al., 2017). RALF34 binds the ECD of ANX1/2 and BUPS1/2 by competing with RALF4/19. The interaction between RALF34 and ANX1/2-BUPS1/2 shut off RALF4/19 signaling pathways for the maintenance of tube integrity. The model is shown in **Figure 5**. The pollen tube releases sperm cells subsequently (Ge et al., 2017). The same receptor complexes realize different biological processes by simply substituting the autocrine signal with the paracrine signal in the different spatiotemporal windows.

### Gamete fusion and double fertilization

Two sperm cells released from the pollen tube are activated. Plasma membrane adhesion and nuclear fusion of male and female gametes occur consequently. In the process mentioned above, the cysteine-rich peptide EC1 (Egg cell 1) secreted by the egg cell plays an important role (Sprunck et al., 2012). Sprunck et al., 2012 found that EC1 is accumulated and constrained in spherical vesicle-like structures within unfertilized egg cells by observation of EC1-GFP fusion. During double fertilization, once sperm cells arrive, EC1 is detected outside the egg cell, especially in the apical region of the degenerated synergid (Sprunck et al., 2012), where gametes fusion occurs (Hamamura et al., 2012). It was shown quantitatively that the intensity of GFP signal gradually increased toward the egg cell membrane during the interaction and fusion of gametes. There are five EC1-encoding genes in *Arabidopsis* that are specifically expressed in egg cells. The recognition receptors in sperm cells or tubes are unknown. However, under the action of EC1, the potential viral-like Fusogen (HAP2/GCS1 that is related in structure to certain viral fusogens to promote the fusion of gamete cell membranes), was redistributed to the surface of sperm cells to regulate their adhesion and separation. (Sprunck et al., 2012; Cyprys et al., 2019). Gamete membrane fusion is promoted by sperm cell-specific expression of DUF679 membrane proteins, DMP8 and DMP9 (Takahashi et al., 2018; Cyprys et al., 2019).

Multiple pollen tubes are directed into one ovule for fertilization compensation in *hap2^-/-^*, a mutant with gamete fusion disorder (Mori et al., 2006; von Besser et al., 2006; Zhong et al., 2022). However, polyspermy block initiates rapidly after the completion of double fertilization. It is correlated with the clearance or degradation of pollen tube attractants and programmed cell death of the synergids. To prevent the attraction to excessive pollen tubes, the persistent synergid fused with primary endosperm is selectively disorganized during endosperm proliferation. The attractants pre-secreted are rapidly diluted and modified (Maruyama et al., 2015; Sprunck and Dresselhaus, 2015; Motomura et al., 2018). Ethylene signaling is activated by fertilization of egg and leads to nucleus disintegration of the synergid (Maruyama et al., 2015; Sprunck and Dresselhaus, 2015). In *Arabidopsis*, the ethylene-free mutants, knocked out five ethylene biosynthetic 1-aminocyclopropane-1-carboxylate oxidase (ACO) genes by CRISPR/Cas9 technology, were investigated (Li et al., 2022). It was indicated that specific components of ethylene signaling pathways, such as the transcription factors ethylene-insensitive 3 (EIN3) (Abozeid et al., 2017) and EIN3-LIKE 1 (EIL1) (Xiao et al., 2021), but not ethylene itself, are required for directing the death of persistent synergid (Li et al., 2022). It is necessary that the persistent synergid undergoes programmed cell death. It seems that the egg and central cell control independent signaling pathways to eliminate the persistent synergid cell and prevent polytubey fertilization (Pereira et al., 2016a). The molecular mechanism is unclear.

The arrival of the first pollen tube at the ovule leads to nitric oxide accumulation in the filiform apparatus. The process depends on de-esterified pectin that is mediated and maintained by FER. Nitric oxide nitrosates the precursor and the mature forms of the attractant AtLURE1 to impede AtLURE1 secretion and interaction with its receptors, and inhibit additional pollen tubes from entering the ovule for polytubey block (Duan et al., 2020). In addition to nitrosation of chemoattractants, AtLURE1s are also digested by egg cell-specific peptidases. The aspartate endopeptidases, EGG CELL-SPECIFIC1/2 (ECS1/2), are expressed specifically in egg cells. The transcripts are degraded immediately after gamete fusion. Once fertilization is completed, ECS1 and ECS2 are secreted from the cortical network at the apical region of *Arabidopsis* egg cell into extracellular space or to the persistent synergid, where it cleaves exclusively and decomposes AtLURE1 (Yu et al., 2021b). The clearing mechanism of XIUQIUs has yet to be discovered. If double fertilization fails, pollen tube attractors will be secreted from the persistent synergid to attract the second pollen tube for fertilization.

## Conclusion remarks

In flowering plants, the cell-cell communication and the signaling network dominated by ligands/receptors are most vividly manifested in the process of fertilization. However, many signal pathways governed by ligand-receptor are still unclear. (1) When the pollen tube grows in the transmitting tract, are there peptide signals secreted by female tissue for rapid tube elongation? How can the two distinct signaling pathways of ANX1/2-BUPS1/2 and LRXs be selected by RALF4/19 for regulating the growth and integrity of pollen tubes? (2) Which factors regulate RALF34 to ensure the timely replacement of RALF4/19 to induce pollen tube rupture at the appropriate spatiotemporal window? (3) The arrest of pollen tube growth and synergid burst resulted from recognition of RALF6/7/16/36/37 by the FER-ANJ-HERK1 receptor. Whether FER-ANJ-HERK1 receives ligand signals secreted by synergid to replace RALF6/7/16/36/37 derived from pollen tubes, triggers the rupture of synergid cells that received pollen tubes? (4) What are the cognate receptors of XIUQIUs during micropylar guidance for pollen tubes? Is there a class of highly conserved receptors that recognize XIUQIUs during their evolution? (5) The EC1 secreted by the egg cell directs the sperm cells to be released at the cleft between the egg and central cell, and promotes gamete fusion and double fertilization. So, does EC1 have a cognate receptor of sperm cells?

With further advances in study, more signaling pathways directly provoked during the plant reproductive process will be discovered and investigated. However, due to the functional redundancy and homology in the genome, it is not easy to identify the phenotype of mutation in a homologous gene. It is suggested that unexpected difficulties in discovering ligands and receptors may be inevitable. Significant progress has been made since the application of CRISPR-Cas9 technology in knocking out multiple homologous genes simultaneously, and obtaining relevant functional mutants is greatly favored as a consequence. It is imperative to develop technologies for high-throughput protein expression and localization. It is expected to integrate high-throughput protein localization and efficient CRISPR-Cas9 knockout technology to leverage forward genetics and elucidate the molecular mechanism of functionally redundant receptor kinases and small peptide ligands in the future. Undoubtedly, the network for cell-cell communication will be dissected with the aid of the coming biotechnologies and ideas.

## Author contributions

C-XX, W-JL, and BW performed the literature search and data analysis. C-XX and T-YY prepared the figure. T-YY drafted and revised the manuscript. All authors contributed to the article and approved the submitted version.
